# Recipient *ADAMTS13* Single-Nucleotide Polymorphism Predicts Relapse after Unrelated Bone Marrow Transplantation for Hematologic Malignancy

**DOI:** 10.3390/ijms20010214

**Published:** 2019-01-08

**Authors:** Haruka Nomoto, Akiyoshi Takami, J. Luis Espinoza, Makoto Onizuka, Koichi Kashiwase, Yasuo Morishima, Takahiro Fukuda, Yoshihisa Kodera, Noriko Doki, Koichi Miyamura, Takehiko Mori, Shinji Nakao, Eriko Morishita

**Affiliations:** 1 Department of Clinical Laboratory Science, Kanazawa University School of Medical Sciences, Kanazawa 920-0942, Japan; haruka84@stu.kanazawa-u.ac.jp (H.N.); eriko86@staff.kanazawa-u.ac.jp (E.M.); 2 Division of Hematology, Department of Internal Medicine, Aichi Medical University School of Medicine, Nagakute 480-1195, Japan; 3 Hematopoietic Cell Transplantation Center, Aichi Medical University Hospital, Nagakute 480-1195, Japan; ykodera@river.ocn.ne.jp; 4 Department of Hematology and Rheumatology, Faculty of Medicine, Kindai University, Osaka 589-8511, Japan; luis1.esp2.cd@outlook.com; 5 Department of Hematology and Oncology, Tokai University School of Medicine, Isehara 259-1193, Japan; moni5@mac.com; 6 Japanese Red Cross Kanto-Koshinetsu Block Blood Center, Tokyo 135-8521, Japan; k-kashiwase@ktks.bbc.jrc.or.jp; 7 Department of Hematology and Cell Therapy, Aichi Cancer Center Hospital, Nagoya 464-8681, Japan; ymorisim@aichi-cc.jp; 8 Hematopoietic Stem Cell Transplantation Unit, National Cancer Center Hospital, Tokyo 104-0045, Japan; tafukuda@ncc.go.jp; 9 Hematology Division, Tokyo Metropolitan Cancer and Infectious Diseases Center Komagome Hospital, Tokyo 113-8677, Japan; n-doki@cick.jp; 10 Department of Hematology, Japanese Red Cross Nagoya First Hospital, Nagoya 453-8511, Japan; miyamu@nagoya-1st.jrc.or.jp; 11 Division of Hematology, Department of Medicine, Keio University School of Medicine, Tokyo 160-8582, Japan; tmori@a3.keio.jp; 12 Hematology/Respiratory Medicine, Faculty of Medicine, Institute of Medical Pharmaceutical and Health Sciences, Kanazawa University, Kanazawa 920-8641, Japan; snakao8205@staff.kanazawa-u.ac.jp

**Keywords:** ADAMTS13, unrelated donor, bone marrow transplantation, single nucleotide polymorphism

## Abstract

Relapse remains a major obstacle to the survival of patients with hematologic malignancies after allogeneic hematopoietic stem cell transplantation. A disintegrin-like and metalloprotease with a thrombospondin type 1 motif (ADMATS13), which cleaves von Willebrand factor multimers into less active fragments, is encoded by the *ADAMTS13* gene and has a functional single-nucleotide polymorphism (SNP) rs2285489 (C > T). We retrospectively examined whether *ADAMTS13* rs2285489 affected the transplant outcomes in a cohort of 281 patients who underwent unrelated human leukocyte antigen (HLA)-matched bone marrow transplantation for hematologic malignancies. The recipient *ADAMTS13* C/C genotype, which putatively has low inducibility, was associated with an increased relapse rate (hazard ratio [HR], 3.12; 95% confidence interval [CI], 1.25–7.77; *P* = 0.015), resulting in a lower disease-free survival rate in the patients with a recipient C/C genotype (HR, 1.64; 95% CI, 1.01–2.67; *P* = 0.045). Therefore, *ADAMTS13* rs2285489 genotyping in transplant recipients may be a useful tool for evaluating pretransplantation risks.

## 1. Introduction

Allogeneic hematopoietic stem cell transplantation (allo-HSCT) has curative potential for patients with hematologic malignancies. However, life-threatening complications, such as severe infections, organ damage and graft-versus-host disease (GVHD), may develop eventually. In particular, disease relapse remains a major drawback due to the subsequent dismal outcomes as it accounts for 20–50% of the primary causes of death associated with allo-HSCT [1,2,3]. Although disease characteristics and HLA matching are the major determinants of a survival outcome after allo-HSCT, other factors, such as sex and its matching of recipients and donors, age of donors, pretransplantation cytomegalovirus (CMV) serostatus and genetic polymorphisms other than HLA, have also been suggested as predictors of the post-transplant survival [4,5,6,7,8]. A better understanding of these predictors for each patient may facilitate personalized adaptability and procedures for allo-HSCT.

A disintegrin-like and metalloprotease with thrombospondin type 1 motif (ADMATS13) produced by vascular endothelial cells, hepatic stellate cells, platelets and kidney podocytes is a protease that can cleave von Willebrand factor (vWF) multimers, thereby governing platelet aggregation and inflammation [9]. A low level of ADAMTS13 activity is known to be associated with not only thrombotic microangiopathy (TMA) but also cancer metastasis, the diagnosis of acute leukemia and disease relapse after allo-HSCT [10,11,12,13]. ADAMTS13 is encoded by the *ADAMTS13* gene on chromosome 9q34 and has one important single-nucleotide polymorphism (SNP) rs2285489 (C > T) located in an intronic region that is functional, high (>0.3) in the minor allele frequency irrespective of the populations and not linked to any other SNPs [14]. The major allele (C) of the *ADAMTS13* rs2285489 SNP has been shown to be associated with lower serum levels of ADAMTS13 than the minor allele (T).

We therefore hypothesized that the *ADAMTS13* rs2285489 SNP might be associated with disease relapse after allo-HSCT. To test this hypothesis, we investigated the influence of the *ADAMTS13* rs2285489 SNP on transplant outcomes in a cohort of patients undergoing unrelated HLA-matched bone marrow transplantation (BMT) for hematologic malignancies through the Japan Marrow Donor Program (JMDP).

## 2. Methods

### 2.1. Patients

ADAMTS13 genotyping was performed on 281 transplantation recipients, including 165 with acute myeloid leukemia (AML) (55.9%), 65 with acute lymphoblastic leukemia (ALL) (23.1%) and 51 with myelodysplastic syndrome (MDS) (18.1%), and their unrelated donors who underwent BMT through the JMDP with T cell-replete marrow from HLA-A, HLA-B, HLA-C, HLA-DRB1, HLA-DQB1 and HLA-DPB1 allele-matched donors between January 2006 and December 2009 (Table 1). The study cohort included only Asian patients and excluded those with any history of transplantation. The final clinical data analyses of these patients were completed by 1 September 2014.

The recipients were defined as having high-risk disease if they had AML or ALL during the first complete remission. All other patients were designated as having standard-risk disease. The conditioning regimen varied according to the underlying disease and the condition of the patient. The combination of cyclophosphamide (CY) combined with total body irradiation (TBI) was mainly used for the myeloablative conditioning (MAC) regimen, whereas the combination of fludarabine and melphalan or busulfan was mainly used for the reduced-intensity conditioning (RIC) regimen [15]. Cyclosporine or tacrolimus with short-term methotrexate was used for GVHD prophylaxis [16,17]. No patients received anti-T cell therapy, such as antithymocyte globulin or ex vivo T cell depletion, in this study.

All patients and donors gave their informed consent at the time of transplantation to take part in molecular studies of this nature, which was carried out in accordance with the Declaration of Helsinki. This project was approved by the Institutional Review Board of Aichi Medical University School of Medicine and the JMDP. All methods were performed in accordance with the approved guidelines and regulations.

### 2.2. Genotyping

Real-time polymerase chain reaction (PCR) genotyping of *ADAMTS13* was performed using the TaqMan-Allelic discrimination method as previously described [7]. The genotyping assay was conducted in 96-well PCR plates using specific TaqMan probes for the *ADAMTS13* gene SNP rs2285489 (C___3183341_20) in a StepOnePlus Real-time PCR system (Applied Biosystems, Foster City, CA, USA).

### 2.3. Data Management and Statistical Analyses

Data were collected by the JMDP using a standardized report form. Follow-up reports were submitted after 100 days, 1 year and then annually after transplantation. Only recipients were routinely measured for the pretransplantation CMV serostatus. The time to neutrophil engraftment was defined as the first of three consecutive days with an absolute neutrophil count of more than 0.5 × 10^9^/L. Acute GVHD developing within the first 100 days post-transplantation was diagnosed and graded based on the established criteria [18]. The classification of chronic GVHD observed in patients who survived beyond day 100 was based on the Seattle criteria [19]. The overall survival (OS) was calculated from the date of transplantation to the date of death from any cause. Disease relapse was defined as the number of days from transplantation to disease relapse or progression. The non-relapse mortality (NRM) was defined as death due to any cause other than relapse or disease progression. The disease-free survival (DFS) was defined as the survival without disease relapse or progression. Any patients who were alive at the last follow-up date were censored. Data regarding the clinical and microbiological characteristics of infections, postmortem changes, prophylaxis against infections and therapy for GVHD given on an institutional basis were not considered in this study.

All of the statistical analyses were carried out using the EZR software package [20]. The probabilities of the OS and DFS were calculated using the Kaplan–Meier method and the comparisons between groups were performed via the log-rank test. The occurrence of NRM, disease relapse, acute GVHD and chronic GVHD were compared using the Gray test [21] and analyzed using the cumulative incidence analysis [22] with relapse, death without disease relapse, death without acute GVHD, death without chronic GVHD and death without engraftment taken into consideration as separate competing risks. A multivariate Cox model was constructed for the OS and DFS while a Fine-Gray competing risk regression model was constructed for NRM, relapse, acute GVHD and chronic GVHD to evaluate the hazard ratio (HR) associated with the *ADAMTS13* genotypes. Recipient characteristics (age and performance status [PS] at the time of BMT, sex, pretransplantation CMV serostatus, disease and disease risk at transplantation), donor characteristics (age, sex, sex compatibility and ABO compatibility), transplant characteristics (MAC or RIC, total number of nucleated cells [TNC] harvested per recipient weight and the use of cyclosporine or tacrolimus as GVHD prophylaxis) and year of transplantation were all used as confounding factors in the multivariate analyses. The Chi-squared and Mann–Whitney tests were used to compare the results of two groups. For all analyses, *P* < 0.05 was considered to be statistically significant.

## 3. Results

### 3.1. Frequencies of ADAMTS13 Genotypes

The frequencies of C/C, C/T and T/T in the *ADAMTS13* rs2285489 C > T were 73.3%, 24.9% and 1.8% among donors and 72.2%, 25.3% and 2.5% among recipients (*P* = 0.801), respectively (Table 1). Since the T/T genotype frequency was low, we decided to compare the C/C genotype and the C/T or T/T genotype in the subsequent analyses.

### 3.2. Transplant Outcomes According to ADAMTS13 Genotypes

**Figure 1:**
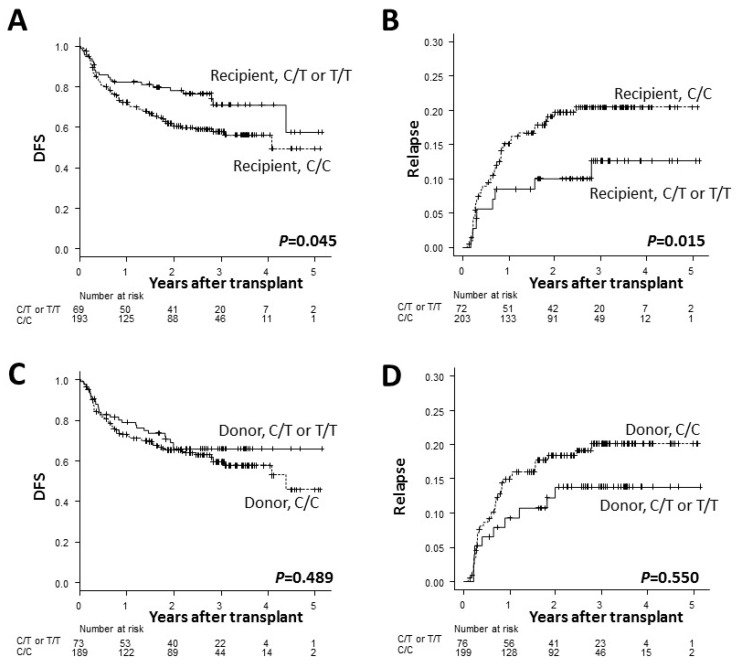
The Kaplan–Meier curves of the adjusted disease-free survival (DFS) rates (**A**,**C**) and the cumulative incidence curves of the adjusted relapse rates (**B**,**D**) after transplantation according to the recipient (**A**,**B**) and donor (**C**,**D**) *ADAMTS13* genotypes. The solid lines represent the C/T or T/T genotype while the dashed lines represent the C/C genotype.

The transplant outcomes according to the *ADAMTS13* genotype are summarized in Table 2. After adjusting for confounding factors in the multivariate model (Supplementary Table S1), the recipient C/C genotype was found to be associated with a significantly lower 3-year DFS (58% vs. 71%; HR, 1.64; 95% confidence interval [CI], 1.01–2.67; *P* = 0.045; Figure 1A) and a significantly higher incidence of relapse (21% vs. 13%; HR, 3.12; 95% CI, 1.25–7.77; *P* = 0.015; Figure 1B) than the recipient C/T or T/T genotype. These effects were not seen when the donor genotype was examined (Figure 1C,D). Three years was set as the study timepoint according to the median follow-up period among the survivors (median, 825 days; range, 8–1879 days). The proportion of all confounding factors according to the recipient *ADAMTS13* genotype was shown in Table 1, in which no significant differences that would have impaired the reliability of the multivariate analysis were noted. The recipient and donor *ADAMTS13* genotypes were found to have no significant effect on the OS, NRM or GVHD (Table 2). When distinguishing between the recipient C/T and T/T genotypes and comparing them with the recipient C/C genotype, the recipient T/T genotype tended to be associated with a favorable DFS (Supplementary Figure S1). However, it should be noted that only four patients possessed the T/T genotype.

**Figure 2:**
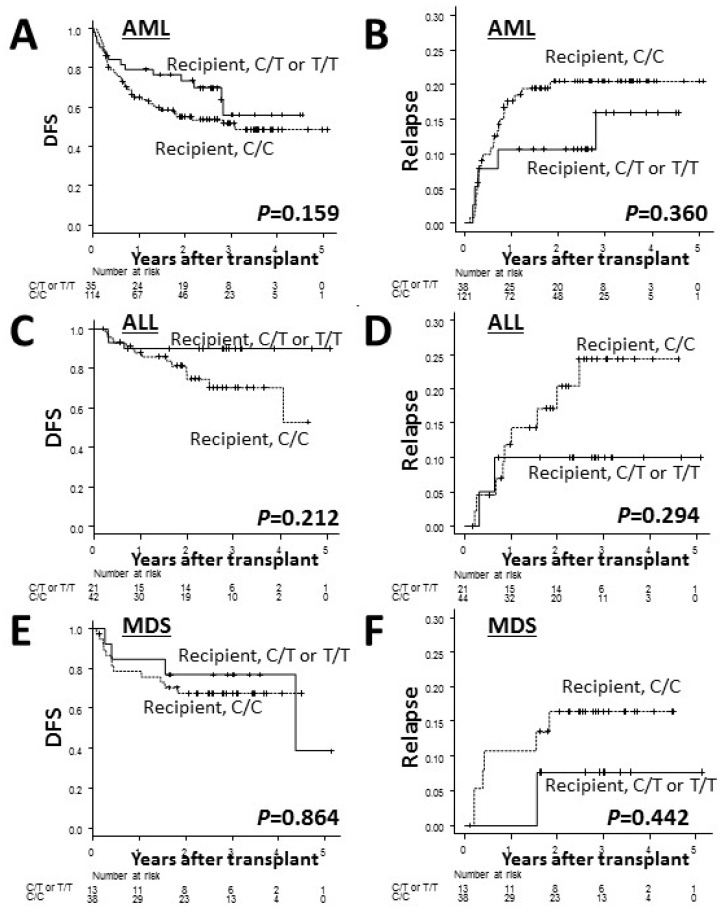
The Kaplan–Meier curves of the adjusted disease-free survival (DFS) rates (**A**,**C**,**E**) and the cumulative incidence curves of the adjusted relapse rates (**B**,**D**,**F**) after transplantation in patients with AML (**A**,**B**), ALL (**C**,**D**) and MDS (**E**,**F**) according to the recipient *ADAMTS13* genotype. The solid lines represent the C/T or T/T genotype while the dashed lines represent the C/C genotype.

When conducting analyses separately for each disease, the recipient C/C genotype showed a lower adjusted DFS and higher relapse rates than the recipient C/T or T/T genotype although no significant difference was found (Figure 2).

**Figure 3:**
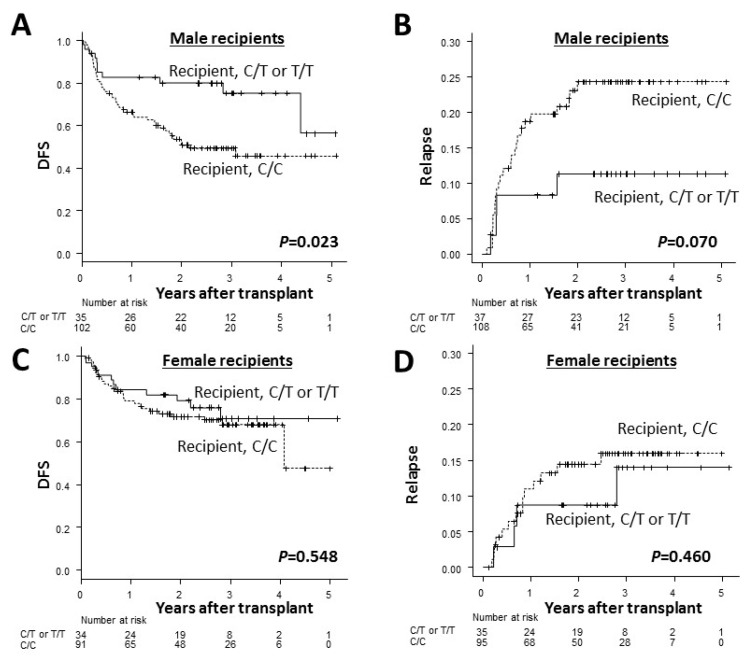

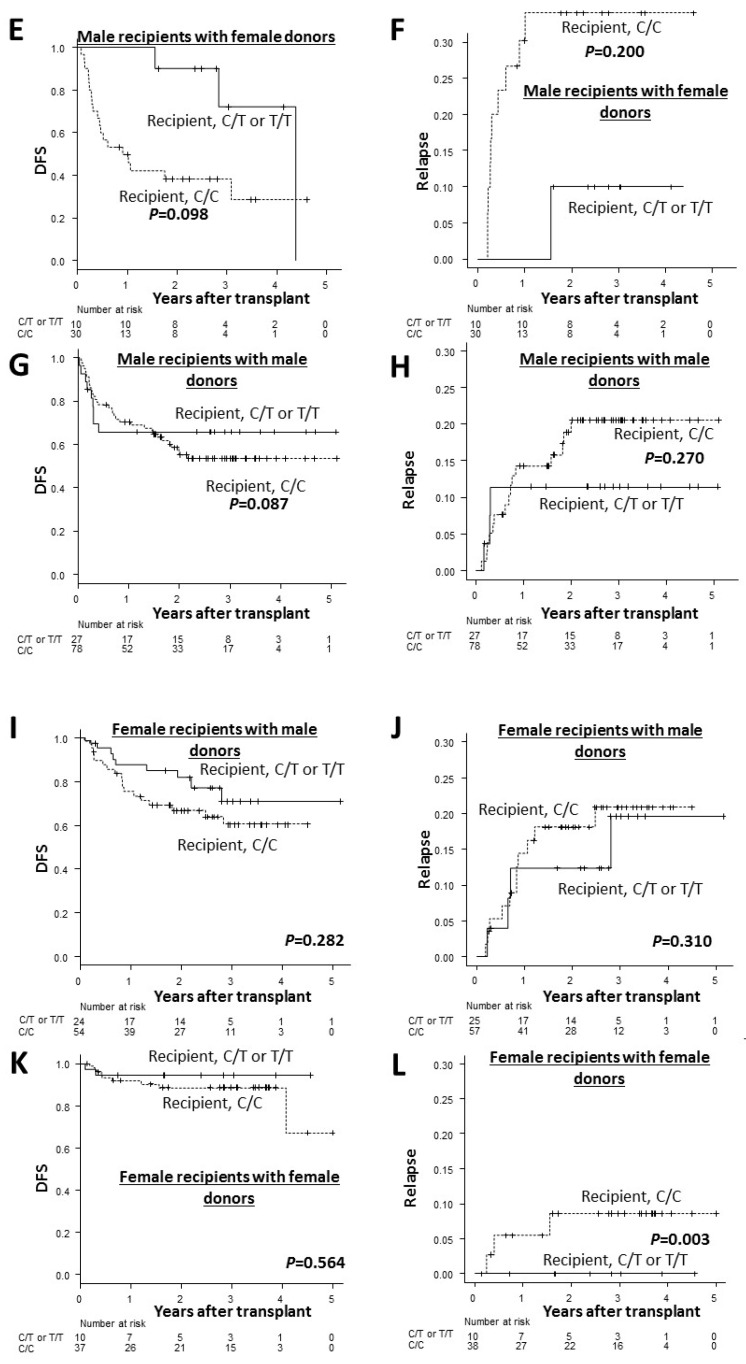
The Kaplan–Meier curves of the adjusted disease-free survival (DFS) rates (**A**,**C**,**E**,**G**,**I**,**K**) and the cumulative incidence curves of the adjusted relapse rates (**B**,**D**,**F**,**H**,**J**,**L**) after transplantation in male recipients (**A**,**B**), female recipients (**C**,**D**), male recipients with female donors (**E**,**F**), male recipients with male donors (**G**,**H**), female recipients with male donors (**I**,**J**) and female recipients with female donors (**K**,**L**) according to the recipient *ADAMTS13* genotype. The solid lines represent the C/T or T/T genotype while the dashed lines represent the C/C genotype.

Since recipient sex and sex matching between recipients and donors had a significant influence on the DFS and relapse rates (Supplementary Table S1), a subgroup analysis was performed with a focus on each recipient’s sex and sex matching between recipients and donors. Figure 3 and Supplementary Table S2 show the adjusted DFS and relapse rates according to the recipient *ADAMTS13* genotype in each subgroup. In male recipients, the recipient C/C genotype adversely affected the DFS and relapse rate (Figure 3A,B), with this same trend found in the DFS results, regardless of sex mismatches with donors (Figure 3E,G). In contrast, the adverse effects of the recipient C/C genotype on the DFS and relapse rate were not evident in female recipients as a whole (Figure 3C,D). However, when the data were analyzed further by the presence or absence of sex mismatches with donors, the recipient C/C genotype tended to result in a lower DFS and higher relapse rate relative to the recipient C/T and C/T genotypes in both groups (Figure 3J,L), particularly with regard to the relapse rate among female recipients with male donors.

## 4. Discussion

The current study showed that the recipient *ADAMTS13* rs2285489 C/C genotype, which putatively has lower inducibility of ADAMTS13 than the C/T or T/T genotype [14], was associated with an increased rate of relapse, leading to a lower DFS in patients with hematologic malignancies receiving unrelated BMT than the recipient C/T or T/T genotype.

The mechanism through which the recipient *ADAMTS13* rs2285489 C/C genotype exerts its detrimental effects remains unclear. A recent report [14] demonstrated that the *ADAMTS13* rs2285489 was functional and the *ADAMTS13* major allele (C) was associated with a reduced ADAMTS13 activity, increasing the risk of pediatric stroke compared with its minor allele (T). The lower translational activity associated with the *ADAMTS13* rs2285489 C/C genotype in hepatic stellate cells, vascular endothelial cells and kidney podocytes might contribute to an increased incidence of relapse. Evidence of a link between the lower ADAMTS13 activity and the higher relapse rate after allo-HSCT was provided in a recent study [13], which showed that lower ADAMTS13 levels before allo-HSCT predicted higher relapse rates after transplantation in AML patients. This hypothesis may also be supported by the decreased ADAMTS13 levels in AML and ALL patients compared to those in normal individuals [11,12]. Furthermore, it is suggested that endothelial dysfunction due to the increased vWF activity may be related to the progress of acute leukemia [23,24]. Therefore, the reduced ADAMTS13 activity associated with the recipient C/C genotype may result in increased vWF, possibly promoting the progression of hematologic malignancies due to a decreased function of vascular endothelial cells, although these considerations remain speculation because the blood vWF levels were not measured in the current study. Furthermore, it is still unclear whether the reduced risk of relapse associated with the *ADAMTS13* minor allele (T) was caused by its anti-malignancy effect or by its induction of graft-versus-malignancy effects. Clarifying these mechanisms may lead to the development of specific anti-hematologic-malignancy therapies and prophylaxis.

The current multivariate analyses showed that the male recipient and sex mismatch between recipients and donors were risk factors for the DFS and relapse rate as well as the recipient *ADAMTS13* C/C genotype (Supplementary Table S1). These findings are consistent with previous reports [4,5], in which male recipients with female donors were associated with a lower survival rate after allo-HSCT for hematologic malignancies. Furthermore, in a recent large-scale retrospective study [6], male recipients were associated with a reduced DFS and increase relapse rate compared to female recipients, independent of the donor sex. Our subgroup analyses according to the recipient sex and sex mismatch showed that adverse effects of the recipient C/C genotype on the DFS and relapse rate were consistently observed in male recipients. However, these effects were not apparent in female recipients, except for the relapse rate in female recipients with female donors (Figure 3L). The reason why the effects of the *ADAMTS13 SNP* on the survival outcomes were not found in the female recipients is unknown. A recent study [25] has reported that blood levels of ADAMTS13 antigens were higher in females than in males, which may suggest that female recipients have a greater amount of ADAMTS13 antigen in the blood and are less likely to be affected by the *ADAMTS13* SNP than male recipients, although this is highly speculative. Further studies will be needed to clarify whether the ADAMTS13 antigen can effectively protect transplant recipients against relapse.

Three major limitations associated with the present study warrant mention. One limitation is that the functional roles of the *ADAMTS13* SNP in relapse after allo-HSCT remain speculative due to the lack of data from the blood samples of allo-HSCT patients and their donors. Likewise, this study did not determine whether there was an association between the serum concentrations of ADAMTS13 and the *ADAMTS13* SNP. The second limitation is that detailed information on sinusoidal obstruction syndrome (SOS)/veno-occlusive syndrome (VOD), such as its incidence and treatment outcomes, was not available in the present study. Furthermore, the effects of the recipient genotypes on the development and treatment outcome of SOS/VOD were unclear, although previous reports [26,27] have suggested an association of a low ADAMTS13 activity with the development of SOS/VOD. Finally, a validation cohort study was not performed to ascertain the current observations because it was beyond the scope of the study, making this an urgent issue to address in the future.

In conclusion, the findings of the present data suggested that the recipient *ADAMTS13* genotype predicted the relapse rate and survival outcome after allo-HSCT from unrelated donors. Therefore, *ADAMTS13* genotyping in transplant recipients may be a useful tool for evaluating the pretransplantation risks that can form a basis for appropriately tailoring transplantation strategies when combined with other currently known risk factors. If *ADAMTS13* genotype information can be used to identify patients prone to relapse in advance, appropriate prophylactic measures and preemptive treatment can be performed prior to hematologic relapse, which may lead to better survival outcomes. Even treatment alternatives other than allo-HSCT should be considered. Aside from HLA and killer-cell immunoglobulin-like receptors [28,29,30], genetic variations that affect the survival outcomes by influencing relapse are rare. Given the plausible functional roles of this SNP, the current findings suggest the pivotal role of ADAMTS13 in disease progression, which may contribute to the development of novel prophylactic and therapeutic strategies for relapse, such as alterations in the in vivo regulation of the *ADAMTS13* gene. Further studies are warranted to ascertain whether or not the findings of this study can be expanded to other stem cell sources or to HLA-mismatched transplantation and to validate the present data in other ethnic groups.

## Figures and Tables

**Table 1:** Recipient and donor characteristics according to the recipient *ADAMTS13* genotype.

	Recipient *ADAMTS13* Genotype, *n* (%)			
Variable	Total	C/C, 206 (73.3%)	C/T, 70 (24.9%) T/T, 5 (1.8%)	*P*
Number of cases	281	206	75	
Recipient age, years, median (range)	47 (1–66)	47 (2–66)	46 (1–65)	0.874
Donor age, years, median (range)	34 (20–66)	34 (20–66)	34 (21–55)	0.582
Year of HSCT, median (range)	2008 (2006–2009)	2008 (2006–2009)	2008 (2006–2009)	0.787
Donor *ADAMTS13* genotype, *n* (%)				
C/C	203 (72.2)	152 (73.8)	51 (68.0)	0.447
C/T	71 (25.3)	50 (24.3)	21 (28.0)	
T/T	7 (2.5)	4 (1.9)	3 (4.0)	
Patient sex, *n* (%)				
Male	150 (53.4)	111 (53.9)	39 (52.0)	0.789
Female	131 (46.6)	95 (46.1)	36 (48.0)	
Donor sex, *n* (%)				
Male	193 (68.7)	138 (67.0)	55 (73.3)	0.383
Female	88 (31.3)	68 (33.0)	20 (26.7)	
Recipient/Donor sex match, *n* (%)				
Sex-matched	158 (56.2)	119 (57.8)	39 (52.0)	0.416
Not sex-matched	123 (43.8)	87 (42.2)	36 (48.0)	
Disease, *n* (%)				
AML	165 (55.9)	124 (60.2)	41 (54.7)	0.509
ALL	65 (23.1)	44 (21.4)	21 (28.0)	
MDS	51 (18.1)	38 18.4)	13 (17.3)	
Disease stage, *n* (%)				
Standard risk	198 (70.5)	148 (71.8)	50 (66.7)	0.460
High risk	83 (29.5)	58 (28.2)	25 (33.3)	
ABO matching, *n* (%)				
ABO-matched	157 (55.9)	112 (54.4)	45 (60.0)	0.641
Major mismatch	74 (26.3)	53 (25.7)	21 (28.0)	0.756
Minor mismatch	69 (24.6)	55 (26.7)	14 (18.7)	0.155
Bidirectional	19 (6.8)	14 (6.8)	5 (6.7)	1.000
Conditioning regimen, *n* (%)	69 (25.1)	57 (28.1)	12 (16.7)	
Reduced intensity	209 (74.4)	147 (71.4)	62 (82.7)	0.064
Myeloablative	72 (25.6)	59 (28.6)	13 (17.3)	
GVHD prophylaxis, *n* (%)				
Cyclosporine	66 (23.5)	54 (26.2)	12 (16.0)	0.082
Tacrolimus	215 (76.5)	152 (73.8)	63 (84.0)	
PS at transplant, *n* (%)				
2–4	17 (6.0)	12 (5.8)	5 (6.7)	0.748
Pretransplantation CMV serostatus, *n* (%)				
CMV-positive recipient	222 (82.5)	162 (78.6)	60 (80.0)	0.854
Missing	12 (4.3)	10 (4.9)	2 (2.7)	
TNC, ×10^8^/kg, median (range)	2.77 (0.54–8.83)	2.66 (0.77–6.29)	2.91 (0.54–8.83)	0.502

HSCT, hematopoietic stem cell transplantation; AML, acute myeloid leukemia; ALL, acute lymphoblastic leukemia; MDS, myelodysplastic syndrome; GVHD, graft-versus-host disease; PS, performance status; CMV, cytomegalovirus; TNC, total number of nucleated cells harvested.

**Table 2:** The results of a multivariate analysis regarding the association between *ADAMTS13* variations and clinical outcomes after transplantation.

**Variable**	***n***	**Adjusted 3-y DFS**	**HR (95% CI)**	***P***	**Adjusted 3-y OS**	**HR (95% CI)**	***P***	**Adjusted 3-y Relapse**	**HR (95% CI)**	***P***
Overall	281	61%			63%			18%		
Recipient *ADAMTS13*, C/C	206	**58%**	**1.64 (1.01–2.67)**	**0.045**	61%	1.40 (0.86–2.29)	0.180	**21%**	**3.12 (1.25–7.77)**	**0.015**
Recipient *ADAMTS13*, C/T or T/T	75	**71%**			71%			**13%**		
Donor *ADAMTS13*, C/C	203	60%	1.18 (0.74–1.89)	0.489	62%	1.23 (0.76–2.01)	0.403	20%	1.26 (0.60–2.66)	0.550
Donor *ADAMTS13*, C/T or T/T	78	66%			67%			14%		
**Variable**	**n**	**Adjusted 3-y NRM**	**HR (95% CI)**	***P***	**Adjusted grades 2–4 acute GVHD**	**HR (95% CI)**	***P***	**Adjusted chronic GVHD**	**HR (95% CI)**	***P***
Overall	281	23%			34%			28%		
Recipient *ADAMTS13*, C/C	206	23%	1.03 (0.55–1.94)	0.920	35%	1.08 (0.64–1.81)	0.770	28%	1.00 (0.57–1.73)	0.990
Recipient *ADAMTS13*, C/T or T/T	75	25%			32%			27%		
Donor *ADAMTS13*, C/C	203	24%	1.03 (0.55–1.91)	0.930	34%	0.93 (0.58–1.49)	0.750	27%	0.74 (0.46–1.22)	0.240
Donor *ADAMTS13*, C/T or T/T	78	22%			34%			33%		

DFS, disease-free survival; HR, hazard ratio; OS, overall survival; CI, confidence interval; NRM, non-relapse mortality. Bolded results regarding the genotype represent *P* < 0.05.

## Supplementary Materials

Supplementary materials can be found at http://www.mdpi.com/1422-0067/20/1/214/s1.
