# Supplementary material for: Recipient ADAMTS13 Single-Nucleotide Polymorphism Predicts Relapse after Unrelated Bone Marrow Transplantation for Hematologic Malignancy
Source: Int J Mol Sci. 2019 Jan 8;20(1):214. doi: 10.3390/ijms20010214 (PMC6337246; doi:10.3390/ijms20010214)
Supplement: Supplementary file 1 [file ijms-20-00214-s001.pdf]

Supplementary Materials

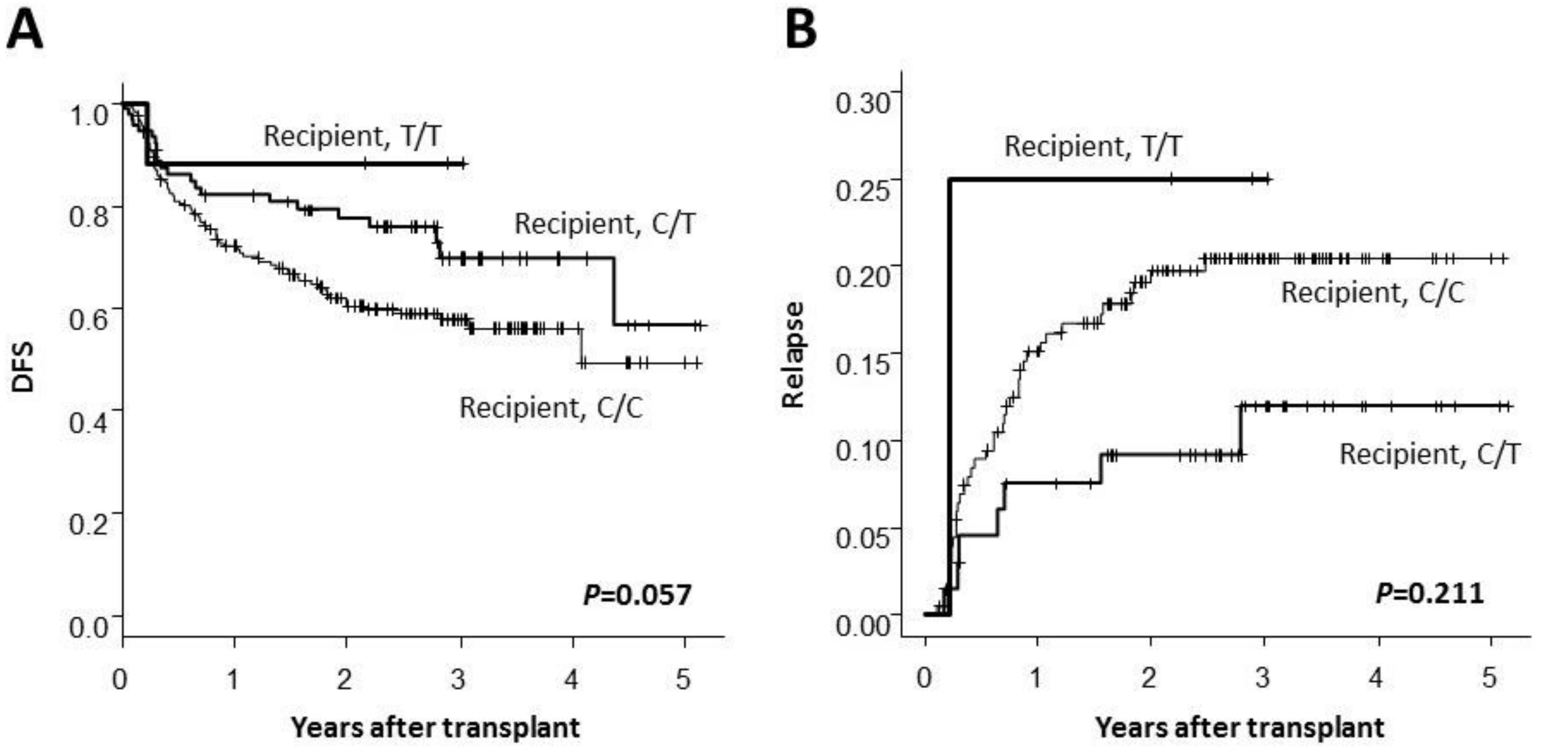

Figure S1. The Kaplan-Meier curves of the adjusted disease-free survival (DFS) rates (**A**) and the cumulative incidence curves of the adjusted relapse rates (**B**) after transplantation according to the recipient *ADAMTS13* genotype.The bold lines represent the T/T genotype, the middle lines represent the C/T genotype, and the thin lines represent the C/C genotype.

Table S1. The results of the multivariate analysis of the association of the recipient *ADAMTS13* genotype with the DFS and relapse incidence after transplantation.

	DFS			Relapse		
Variable	HR	95% CI	*P*	HR	95% CI	*P*
Recipient *ADAMTS13*, C/C *vs*. C/T or T/T	1.64	1.01-2.67	0.045	3.12	1.25-7.77	0.015
Donor *ADAMTS13*, C/C *vs*. C/T or T/T	1.18	0.74-1.89	0.489	1.26	0.60-2.66	0.550
Donor age	0.99	0.96-1.02	0.398	0.98	0.94-1.02	0.370
Recipient age	1.01	1.00-1.03	0.043	1.00	0.98-1.03	0.660
Donor sex, female *vs.* male	0.98	0.62-1.54	0.932	0.77	0.34-1.77	0.540
Recipient sex, female vs. male	0.53	0.34-0.83	0.006	0.39	0.18-0.87	0.021
Sex mismatch	1.48	0.95-2.31	0.855	3.27	1.37-7.79	0.007
Year of transplant	1.28	0.99-1.65	0.056	0.91	0.49-1.70	0.780
Pretransplantation, recipient CMV status	1.13	0.64-2.02	0.671	1.05	0.40-2.74	0.920
Disease stage, high-risk *vs.* low-risk	2.65	1.76-4.00	<0.001	2.04	1.06-3.91	0.032

MAC *vs.* RIC	1.05	0.65-1.69	0.844	1.05	0.49-2.22	0.910
ABO major mismatch	1.04	0.67-1.63	0.856	1.27	0.66-2.46	0.480
ABO minor mismatch	0.88	0.54-1.44	0.611	1.47	0.71-3.04	0.290
TNC	1.05	0.85-1.29	0.669	1.43	1.09-1.87	0.009
Cyclosporine as GVHD prophylaxis *vs.* tacrolimus	1.02	0.64-1.62	0.952	1.88	1.01-3.50	0.045
PS 2-4	2.15	1.03-4.52	0.043	3.43	1.34-8.79	0.010

CMV, cytomegalovirus; MAC, myeloablative conditioning; RIC, reduced-intensity conditioning; TNC, total number of nucleated cells harvested; GVHD, graft-versus-host disease; PS, performance status.

Table S2. The results of the subgroup analysis by recipient and donor sex regarding the association between recipient *ADAMTS13* variations and survival outcomes after transplantation.

Variab8le	n	Adjusted 3-y DFS	HR (95% CI)	*P*	Adjusted 3-y OS	HR (95% CI)	*P*	Adjusted 3-y relapse	HR (95% CI)	*P*
Male recipient	150	55%			55%			21%		
Recipient *ADAMTS13*, C/C	111	**49%**	**2.22 (1.12-4.41)**	**0.023**	**50%**	**2.02 (1.01-4.02)**	**0.046**	24%	3.93 (0.89-12.30)	0.070
Recipient *ADAMTS13*, C/T or T/T	39	**75%**			**69%**			11%		
Male recipient with female donor	40	46%			51%			28%		
Recipient *ADAMTS13*, C/C	30	38%	3.85 (0.78-18.99)	0.098	45%	4.00 (0.69-23.3)	0.123	34%	4.00 (0.49-32.60)	0.200
Recipient *ADAMTS13*, C/T or T/T	10	72%			71%			10%		

Male recipient with male donor	110	57%			56%			18%		
Recipient *ADAMTS13*, C/C	81	54%	2.07 (0.90-2.78)	0.087	52%	1.95 (0.85-4.47)	0.112	21%	2.84 (0.44-18.28)	0.270
Recipient *ADAMTS13*, C/T or T/T	29	66%			68%			11%		
Female recipient	131	66%			67%			15%		
Recipient *ADAMTS13*, C/C	95	67%	1.25 (0.60-2.62)	0.548	70%	0.98 (0.45-2.10)	0.951	16%	1.70 (0.42-6.85)	0.460
Recipient *ADAMTS13*, C/T or T/T	36	70%			60%			14%		
Female recipient with male donor	83	56%			66%			21%		

Recipient *ADAMTS13*, C/C	57	61%	1.65 (0.66-4.10)	0.282	65%	1.16 (0.43-3.05)	0.777	21%	2.94 (0.36-24.10)	0.310
Recipient *ADAMTS13*, C/T or T/T	26	66%			54%			20%		
Female recipient with female donor	48	76%			78%			7%		
Recipient *ADAMTS13*, C/C	38	89%	1.65 (0.30-8.91)	0.564	91%	1.00 (0.16-6.08)	0.998	**9%**	**599 (8.48-42280)**	**0.003**
Recipient *ADAMTS13*, C/T or T/T	10	94%			94%			**0%**		

DFS, disease-free survival; HR, hazard ratio; OS, overall survival; CI, confidence interval. Bold results regarding the genotype represent *P* <0.05.
